# MiR-1246 regulates the PI3K/AKT signaling pathway by targeting PIK3AP1 and inhibits thyroid cancer cell proliferation and tumor growth

**DOI:** 10.1007/s11010-021-04290-3

**Published:** 2021-12-06

**Authors:** Jingyan Li, Zhanlei Zhang, Jieting Hu, Xiaoting Wan, Wei Huang, Hong Zhang, Ningyi Jiang

**Affiliations:** 1 Department of Nuclear Medicine, Sun Yat-Sen Memorial Hospital, Sun Yat-Sen University, Guangzhou, China; 2 Department of Nuclear Medicine, The Seventh Affiliated Hospital, Sun Yat-Sen University, Guangzhou, China; 3 Guangdong Provincial Key Laboratory of Malignant Tumor Epigenetics and Gene Regulation, Guangzhou, China

**Keywords:** miR-1246, Thyroid cancer, PIK3AP1, PI3K, AKT, Neoplasm metastasis

## Abstract

One of the most prevalent forms of endocrine malignancies is thyroid cancer. Herein, we explored the mechanisms whereby miR-1246 is involved in thyroid cancer. Phosphoinositide 3-kinase adapter protein 1 (PIK3AP1) was identified as a potential miR-1246 target, with the online Gene Expression Omnibus (GEO) database. The binding between miR-1246 and PIK3AP1 and the dynamic role of these two molecules in downstream PI3K/AKT signaling were evaluated. Analysis of GEO data demonstrated significant miR-1246 downregulation in thyroid cancer, and we confirmed that overexpression of miR-1246 can inhibit migratory, invasive, and proliferative activity in vitro and tumor growth in vivo. Subsequent studies indicated that miR-1246 overexpression decreased the protein level of PIK3AP1 and the phosphorylation of PI3K and AKT, which were reversed by PIK3AP1 overexpression. At the same time, overexpression of PIK3AP1 also reversed the miR-1246 mimics-induced inhibition proliferative, migratory, and invasive activity, while promoting increases in apoptotic death, confirming that miR-1246 function was negatively correlated with that of PIK3AP1. Subsequently, we found that the miR-1246 mimics-induced inhibition of PI3K/AKT phosphorylation was reversed by the PI3K/AKT activator IGF-1. miR-1246 mimics inhibited proliferative, migratory, and invasive activity while promoting increases in apoptotic death, which were reversed by IGF-1. Furthermore, miR-1246 agomir can inhibit tumor growth in vivo. We confirmed that miR-1246 affects the signaling pathway of PI3K/AKT via targeting PIK3AP1 and inhibits the development of thyroid cancer. Thus, miR-1246 is a new therapeutic target for thyroid cancer.

## Introduction

One of the most pervasive malignancies of the endocrine system in the USA is thyroid cancer. Thyroid cancer morbidity and mortality have continued to increase in the past decades [1]. Although radioactive iodine is effective in treating thyroid cancer, its rational use and dosage remain controversial [2]. Thyroid cancer develops from follicular and parafollicular cells. More than 90% of thyroid cancer originates from follicular cells, which can absorb iodine to synthesize thyroid hormone [3]. Follicular thyroid carcinoma is the second leading differentiated thyroid carcinoma histological type, with unique biological behavior and poor prognosis, which make it challenging to treat [4]. Different pathological patterns of thyroid cancer have different underlying pathogenesis [5]. Exploring the mechanisms underlying thyroid cancer pathogenesis is critical for designing diagnostic and therapeutic strategies.

MicroRNAs (miRNA) are non-coding RNAs that are  ~ 22 nucleotides long and are widely found in various species, such as animals, plants, and viruses [6]. The development of many cancers has been shown to be inhibited by miRNA overexpression or inhibition [7, 8]. miR-1246 performs an essential task in the oncogenesis of certain cancers, such as colorectal [9], breast cancer [10], and oral carcinomas [11]. However, how miR-1246 functions in thyroid cancer and the underlying mechanism have not been previously reported.

Considerable evidence demonstrates that miRNAs can negatively regulate target messenger RNAs (mRNA) by inhibiting protein translation or inducing mRNA degradation by acting on the 3′-non-translational region (3′-UTR) of target genes [12, 13]. Phosphoinositide 3-kinase adapter protein 1 (PIK3AP1) leads to the activation of phosphatidylinositide 3-kinase (PI3K) through Toll-like receptor (TLR) signaling, preventing excessive inflammatory cytokine production [14]. Meanwhile, studies have revealed that PIK3AP1 plays a vital role in many diseases, like colorectal cancer [15], glioblastoma multiforme [16], and prostate cancer [17]. It is worth noting that there are no reports of miR-1246 targeting PIK3AP1. There are, however, reports showing that PIK3AP1 plays a key role in PI3K/AKT signaling [18–20].

PI3K is an intracellular phosphatidylinositol kinase and AKT (a.k.a. protein kinase B) is a serine/threonine kinase. PI3K/AKT signaling controls the growth of a variety of cells and is involved in processes, such as glucose metabolism [21], apoptosis [22], cell proliferation [23], transcriptional regulation [24], and cell migration [25].

This study sought to investigate the potential of using miR-1246-PIK3AP1-PI3K/AKT-targeted therapy for thyroid cancer.

## Materials and methods

### Gene expression omnibus (GEO) dataset analysis

We downloaded raw gene expression data from the US National Center for Biotechnology Information (NCBI) GEO available online at: https://www.ncbi.nlm.nih.gov/geo/. The samples (filename GSE53072_RAW.tar) were categorized into two groups: the control group comprising data from three normal thyroid glands (GSM1281638, GSM1281639, and GSM1281640) and the thyroid cancer group comprising data from five thyroid carcinoma groups (GSM1281636, GSM1281637, GSM1281641, GSM1281642, and GSM1281643). We performed Affymetrix Human Genome U133 Plus Version 2.0 Array (GPL570) analysis using the Affymetrix Transcriptome Analysis Console (both from Affymetrix, Santa Clara, CA, USA). Differentially expressed miRNAs/mRNAs were considered those that met the following criteria: *P* < 0.05 and log_2_|fold change (FC)|> 1.5. We drew a heatmap and a volcano plot using the results of differentially expressed miRNA/mRNA analysis. We used data from TCGA-THCA to analyze the disregulated mRNAs and the TargetScan database (version 7.2; http://www.targetscan.org/vert_72/) to investigate potential downstream miRNA targets.

### Pathway enrichment analysis

We performed pathway enrichment analysis of differentially expressed mRNAs using the KEGG and the R software package cluster filter (version 3.10.1; https://guangchuangyu.github.io/software/clusterProfiler/).

### Cell culture

The human thyroid cell line KAT18, human papilloma thyroid cancer cell line TPC-1, human follicular thyroid cancer cell line FTC-133 derived from lymph node metastasis of follicular thyroid cancer, human thyroid cancer papillary cell line BCPAP, and Nthy-ori 3–1 control cells were from ProCell (Wuhan, China), and were grown in RPMI-1640 (Gibco, MA, USA) with 10% FBS (Gibco), streptomycin (100U/mL), and penicillin sodium (100U/mL). The culture conditions were 37 °C and 5% CO_2_. IGF-1) treatment, cells were grown in media containing 400 ng/mL IGF-1 (Sigma-Aldrich, MO, USA) at 37 °C for 24 h. Following IGF-1 treatment, the milieu was substituted with fresh milieus. Cells were tested for mycoplasma contamination approximately once a month using the MycoAlert Mycoplasma Detection Kit (Cat. No. LT07-218; Lonza Cologne GmBH, Cologne, Germany).

### Transfection

miR-1246 mimics (50 nM) and it’s negative control (NC; 50 nM) or overexpressed plasmid PIK3AP1 (ov-PIK3AP1; 100 nM) and it’s negative control (ov-NC; 100 nM) were transfected into TPC-1 and FTC-133 cells at 37 °C utilizing Lipofectamine® 2000 (Invitrogen) for 4 h following the instructions of the manufacturer. Their sequence is as follows: miR-1246: 5*′*-AATGGATTTTTGGAGCAGG-3*′*; negative controls (scramble): 5*′*-AGCGTGGTGTGAATGTATA-3*′*. Construction of PIK3AP1 overexpression vector: cDNAs of the PIK3AP1 coding region were cloned into the pcDNA3.1 vector (Invitrogen) using the EcoRI and NotI sites. All RNA and plasmids were prepared through Sangon Biotech Co., Ltd (Shanghai, China).

### Cell proliferation assay

TPC-1 and FTC-133 cells were digested with trypsin, inoculated in 96-well plates (3 × 10^3^/well) for 24–72 h. Absorbance at 450 nm was then assessed every 24 h with 10 μl of the CCK-8 reagent (Solarbio, Beijing, China) was added to cell culture medium every 24 h and incubated at 37 °C for 1 h. Absorbance at 450 nm was assessed to calculate logarithmic growth of cells. The optical density (OD) was measured at 490 nm using an enzyme-labeled instrument (Thermo Fisher Scientific).

### Cell apoptosis assay

The assay of cell apoptosis was performed by applying an Annexin V–FITC Apoptosis Detection Kit (BD Biosciences, CA, USA) based on provided directions. The cells of TPC-1 and FTC-133 were collected, washed two times in cold PBS, resuspended in 500 μL binding buffer, and stained by using 5 μL each of PI and Annexin V–FITC for 15 min at 23 ± 2 °C protected from light. A flow cytometer was then used to assess apoptosis (FCM; BD Biosciences).

### Transwell assay

The invasion and migration of TPC-1 and FTC-133 cells were measured by utilizing 24-well Transwell inserts (BD Biosciences). For the invasion assay, these inserts were initially coated for 6 h using a layer of Matrigel® (BD Biosciences) at 37 °C. Harvested cells (1 × 105) in serum-free milieu were placed in the top chamber, while RPMI-1640 with 10% FBS was added to the lower chamber. After 2 days of incubation, invasive cells were fixed using anhydrous ethanol for 20 min and stained using 0.1% crystal violet (Solarbio) for 15 min at 23 ± 2 °C. Cells were then counted using a light microscope (Olympus Corporation; magnification, × 200).

### RT-qPCR

We extracted total RNA from TPC-1 and FTC-133 cells with TRIzol (Invitrogen) as per provided directions, after which a Kit of PrimeScript RT Reagent (TaKaRa, Dalian, China) was employed to prepare cDNA, and RT-qPCR was conducted utilizing a SYBR Premix ExTaq II kit (Invitrogen) and an ABI 7500 system (Applied Biosystems, CA, USA). The *PIK3AP1* and *GAPDH* primers were: *PIK3AP1*: forward, 5′-TCATCGTCTACAGCCCGGAT-3′, reverse, 5′-TCAGTATCTTCTGGCTGCGG-3′; *GAPDH*: forward, 5′-GCTCATTTGCAGGGGGGAG-3′, reverse, 5′-GTTGGTGGTGCAGGAGGCA-3′. *GAPDH* was used as a reference for calculating *PIK3AP1* expression. The primers for miR-1246 and U6 were as follows: miR-1246: forward, 5′-ACACTCCAGCTGGGAATGGATTTTTGG-3′, reverse, 5′-CTCAACTGGTGTCGTGGA-3′; and U6: forward, 5′-CTCGCTTCGGCAGCACA-3′, reverse, 5′-AACGCTTCACGAATTTGCGT-3′. U6 was employed as an internal reference for calculating the levels of miR-1246. Relative miR-1246 and *PIK3AP1* expression were determined using the 2^−ΔΔCt^ method [26].

### Western blotting

The lysis of TPC-1 and FTC-133 was carried out utilizing chilled lysis buffer (Solarbio), and the protein levels were assessed via BCA assay kit (Solarbio). We separated denatured proteins (20 μg) using 10% SDS-PAGE and transferred them onto membranes of PVDF (Millipore, Sigma-Aldrich). Following the blocking with 5% BSA Blocking Buffer (Solarbio), the incubation of the membranes was carried out at 4 °C during the night hours with the following diluted primary antibodies: PIK3AP1 (1:1000, ab237629, Abcam, Cambridge, UK), AKT (1:500; ab8805; Abcam), p-AKT (1:500, ab38449, Abcam), PI3K (1:1000, #4257, CST, MA, USA), and p-PI3K (1:1000, #4228, CST). After washing thrice in TBST (10 min/wash; Solarbio), blots were probed with secondary HRP-goat-anti-rabbit IgG (1:10,000, ab205718; Abcam) for 2 h at 23 ± 2 °C. Proteins were discovered via improved chemiluminescence (ECL; Thermo Fisher Scientific), and images were obtained with an imaging system (DNR Bio-Imaging Systems Ltd., Mahale HaHamisha, Israel). GAPDH (1:10,000, ab181602, Abcam) was used as the loading control.

### Dual-luciferase reporter assay

The amplified 3′-UTR fragments of PIK3AP1 were cloned into the psi-CHECK-2 vector (Promega Corporation). Subsequently, 293 T cells (2 × 104, CRL-11268, American type culture collection) were transfected with 50 nM miR-1246 mimics or inhibitor, 100 nM of their negative controls, 0.5 µg of psi-CHECK-2 luciferase reporter vector comprising the wild-type (WT) or mutant (Mut) 3′-UTR sequences of PIK3AP1, or empty vector, with Lipofectamine® 2000 (Invitrogen) based upon provided directions. At 48 h post transfection, the luciferase activity was assessed utilizing the Dual-Luciferase Reporter Assay System (GloMax; Promega), with Renilla luciferase activity serving as a normalization control.

### Animal experiments

Twenty-four 8-week-old female BALB/c-nu/nu specific pathogen-free (SPF) mice with a weight of 13–15 g obtained from the Guangdong Medical Laboratory Animal Center (license, No. SCXK, Guangdong, 2016-0041) were used in this study. Mice were placed in a room with a constant temperature and humidity (temperature: 23 ± 2 °C, humidity: 50 ± 10%), under a 12 h cycle of dark/light with free access to standard rat food and water in a polystyrene cage. All animal assessments were confirmed through the Animal Care and Use Committee of Sun Yat-Sen University and conducted conforming to the National Institutes of Health protocols.

The vital characteristics, food intake and body weight of the experimental mice were normal. Following terminal anesthesia using i.p. injection of 3% pentobarbital sodium (100 mg/kg), a subcutaneous tumorigenesis model was established by injecting 1 × 10^6^ TPC-1 and FTC-133 cells transfected with miR-1246 agomir and agomir NC into the subcutaneous side of nude mice. Furthermore, a total of 1 × 106 TPC-1 and FTC-133 cells transfected with miR-1246 agomir and agomir NC were injected into the tail vein of nude mice. Following a 6-week period, an intraperitoneal injection of 3% pentobarbital sodium (200 mg/kg) was used to euthanize these mice. The lung and tumor tissues were quickly stripped and then fixed in 10% neutral formalin solution. Tumor volume was calculated as: Tumor volume = A (maximum diameter) × B (vertical diameter)^2^ × 0.5. miR-1246 agomir and miR-1246 agomir NC (scramble) were synthetic by Ribobio Co., Ltd (Guangzhou, China).

### Hematoxylin and eosin (H&E) staining

Lung tissue was paraffin embedded and sliced into 5 μm thick sections, fixed on a glass slide, and dried. As per provided directions, the sections were stained with the HE staining reagent (Solarbio, Beijing, China). The slices were soaked in xylene and a gradient concentration of ethanol, soaked in hematoxylin, and finally sealed with resin. Subsequently, the lung tissue sections were observed and imaged via light microscope (Olympus, Tokyo, Japan).

### Statistical studies

Data are means ± standard deviation (SD) from triplicate evaluations and were assessed by SPSS 22.0 (IBM Corp., NY, USA). We performed statistical analysis using one-way ANOVAs and Dunnett’s post hoc test. For independent two-group analyses, Student’s *t* tests were used.

## Results

### miR-1246 expression was downregulated in patients with thyroid cancer

**Fig. 1:**
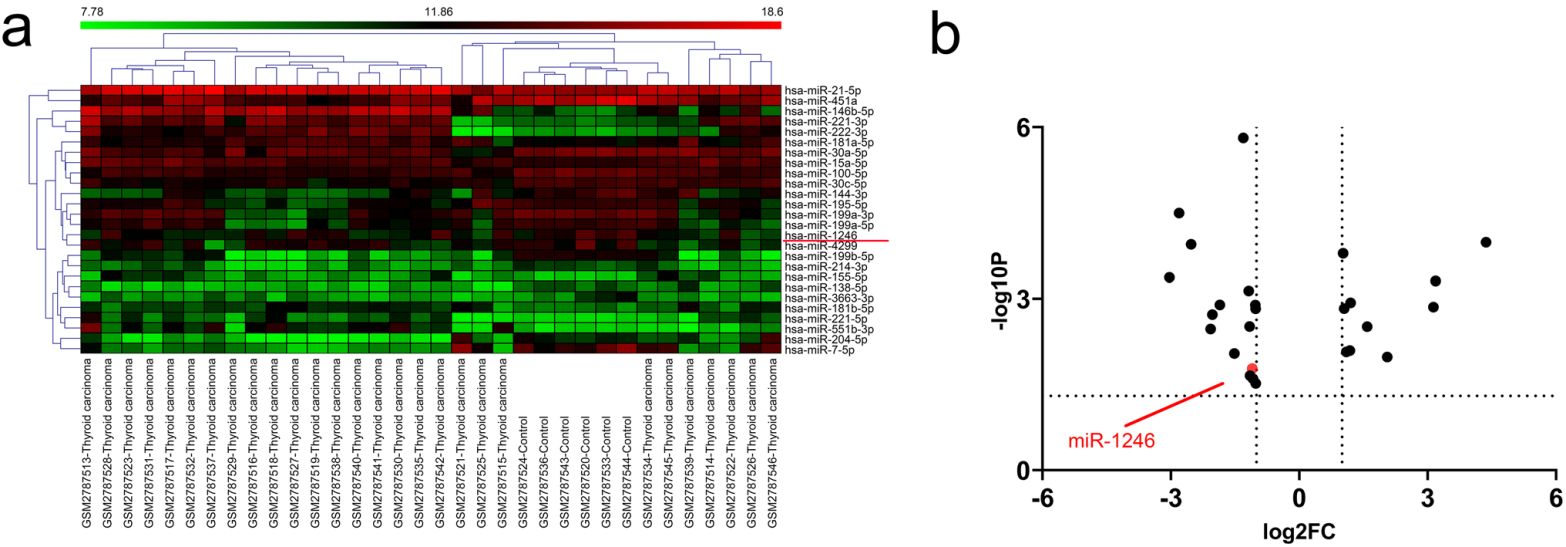
MiR-1246 expression is downregulated in cases with thyroid cancer. **a** Heatmap showing the 26 miRNAs that met the *P* < 0.01 and log_2_|Fc|> 1.5 criteria. **b** Volcano map showing the 26 miRNAs that met the *P* < 0.01 and log2|Fc|> 1.5 criteria; the red dots represent miR-1246 (log_2_Fc < 1.5, low expression)

To screen for miRNA expression differences between thyroid cancer patients and normal controls, we analyzed the GSE53072 dataset from NCBI GEO database. Cluster analysis of the GSE53072 dataset revealed 1541 differentially expressed miRNAs between typical thyroid tissues and human thyroid cancer tissues. Among them, 26 miRNAs showed considerable discrepancies in expression between the two groups (*P* < 0.01 and log_2_|Fc|> 1.5) (Fig. 1a, b). Among these malregulated miRNAs, miR-1246 is considered to be involved in the occurrence and advancement of many cancer [9, 10]. miR-1246 expression was considerably diminished in the samples of thyroid cancer, suggesting that miR-1246 could be correlated with the onset of thyroid cancer and that it could be a promising predictive biomarker for thyroid cancer diagnosis and prognosis. However, its role in thyroid cancer and its potential molecular mechanism have not been determined. Therefore, we focused on further investigating the role of miR-1246 in thyroid cancer.

### miR-1246 expression was downregulated in the cell lines of thyroid cancer

**Fig. 2:**
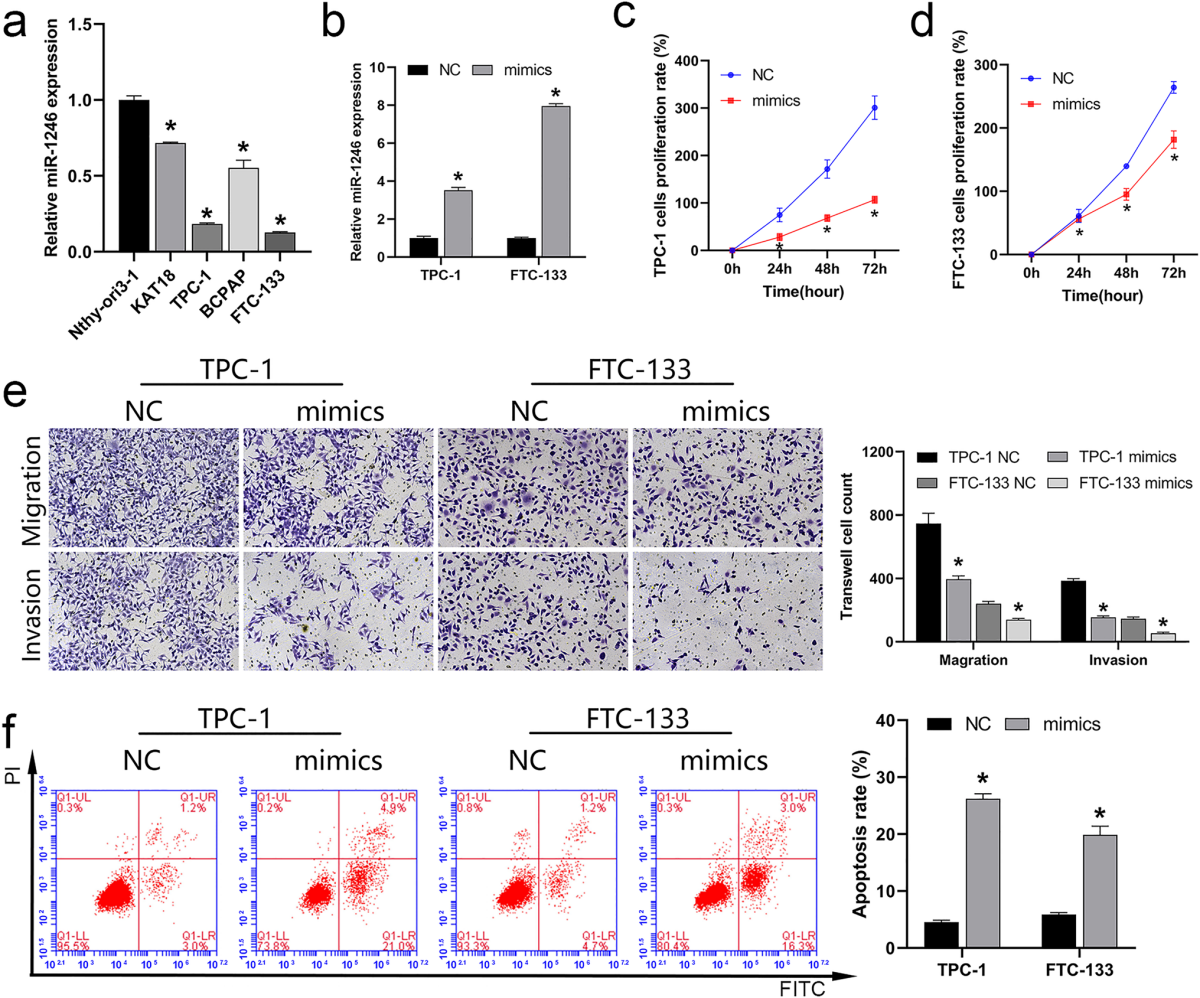
Effect of miR-1246 on cell function of thyroid cancer cell lines. **a** RT-qPCR analysis of miR-1246 expression in the five cell lines. **b** RT-qPCR was utilized to verify the expression of miR-1246 mimics in TPC-1 and FTC-133 cells. **c**, **d** CCK-8 analysis of TPC-1 and FTC-133 cell proliferation. **e** Transwell analysis of migration and invasion of TPC-1 and FTC-133 cell lines (magnification, 200×). **f** Apoptosis level of TPC-1 and FTC-133 cells analyzed using FCM. *P < 0.05. *RT-qPCR* Real-time quantitative reverse transcription polymerase chain reaction, *CCK-8* cell counting kit-8, *FCM* flow cytometry, *NC* negative control

To verify that the expression of miR-1246 was consistent with the results of GEO data analysis, we analyzed miR-1246 levels in the cell lines of thyroid cancer via RT-qPCR, indicating that this miRNA was downregulated in KAT18, TPC-1, FTC-133, and BCPAP cells compared with the levels in the control Nthy-ori 3–1 cell line (Fig. 2a). This confirmed the results of GEO data analysis and indicated that the low expression of miR-1246 may drive thyroid cancer development. It is worth noting that the expression of miR-1246 was the most significant downregulated in TPC-1 and FTC-133 cells, so we selected these two cell lines for future cell function studies to explore the potential mechanism of miR-1246 action.

### miR-1246 overexpression inhibits thyroid cancer cell development

To verify that upregulation of miR-1246 can suppress thyroid cancer cell development, we transfected cells with miR-1246 mimics to overexpress miR-1246. RT-qPCR achievements divulged that the transfection of miR-1246 mimics effectively increased the expression of miR-1246 in the cells of TPC-1 and FTC-133, indicating that the synthetic miR-1246 mimics were effective (Fig. 2b). To examine how miR-1246 may affect thyroid cancer cells, we assessed the impact of miR-1246 mimics on the proliferative, migratory, invasive, and apoptotic activity of these cells. CCK-8 (Fig. 2c, d), transwell (Fig. 2e), and flow cytometry (FCM) (Fig. 2f) results showed that miR-1246 mimics suppressed proliferation, migration, invasion, and drove apoptotic death, suggesting that the upregulation of miR-1246 expression contributes to preventing the development of thyroid cancer cells and improves cell apoptosis.

### miR-1246 directly targeted PIK3AP1 and inhibited PI3K/AK phosphorylation

**Fig. 3:**
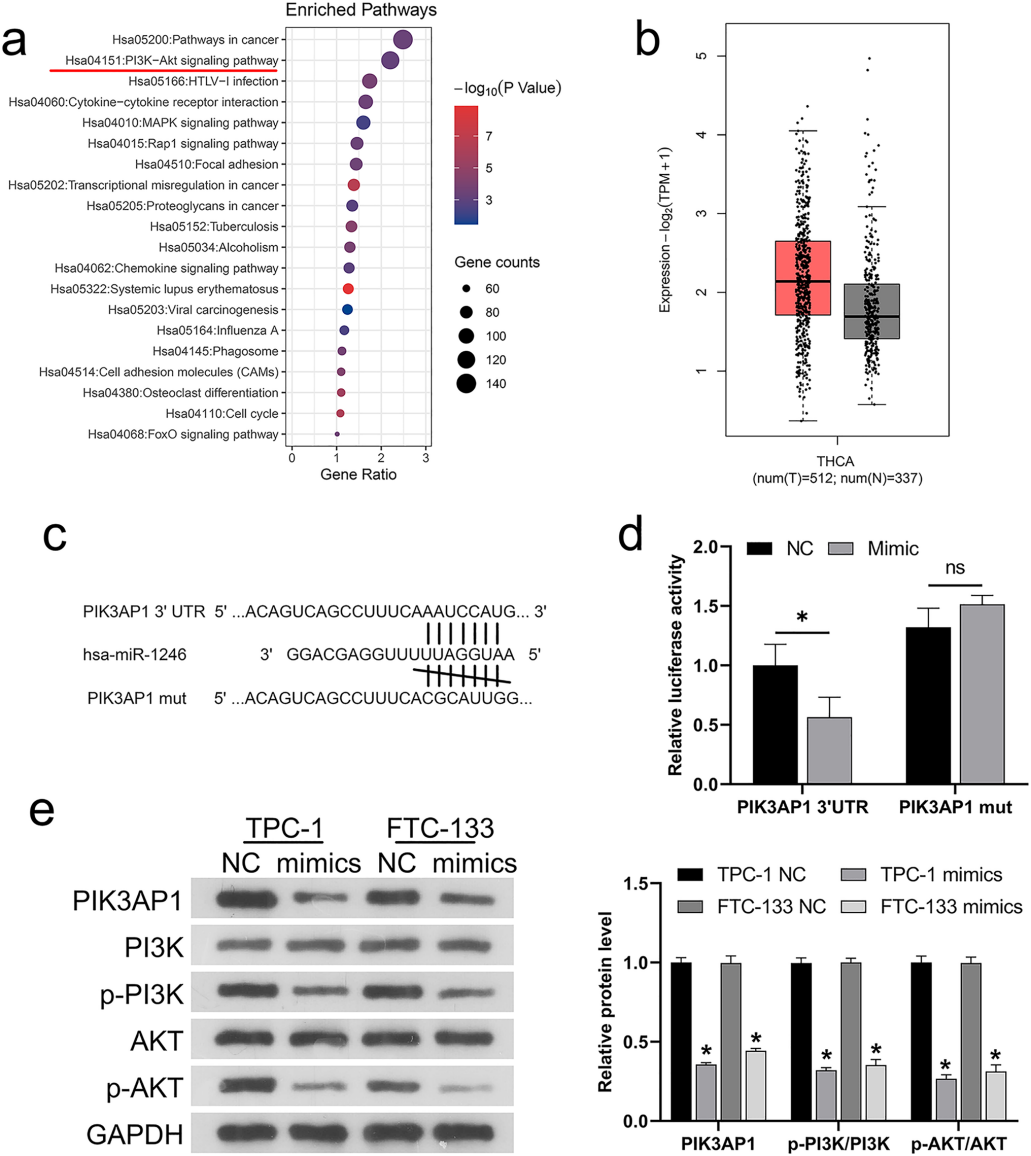
miR-1246 regulates PIK3AP1. **a** KEGG analysis of the Top 20 signaling pathways affecting thyroid cancer development. **b** Boxplot result of TCGA-THCA analysis showing PIK3AP1 upregulation in thyroid cancer. **c** Predicting miR-1246-PIK3AP1 binding sites using the TargetScan Database. **d** Dual-luciferase analysis of miR-1246-PIK3AP1 binding. **e** Western blotting analysis of the protein levels of PIK3AP1, p-PI3K, PI3K, p-AKT, and AKT in TPC-1 and FTC-133 cells. *P < 0.05. *KEGG* Kyoto encyclopedia of genes and genomes, *PIK3AP1* phosphoinositide 3-kinase adapter protein 1, *NC* negative control

miRNAs usually regulate cell function by binding to target genes [27]. From the above results, we know that excessive miR-1246 can suppress the development of thyroid cancer cells, so how does it affect downstream target genes and signaling pathways? Subsequently, KEGG analysis showed a possible role for the signaling pathway of PI3K/AKT in the development of thyroid cancer (Fig. 3a). KEGG analysis also found that many genes may affect the PI3K/AKT pathway, such as MYC, PIK3AP1, COL5A1, and IGF-1. We assessed the putative targets of miR-1246 using the TargetScan database and found that one of the putative target genes, PIK3AP1, had binding sites with miR-1246 and may also be a key gene affecting PI3K/AKT signaling. Meanwhile, PIK3AP1 upregulation was noted in thyroid cancer samples compared with healthy human tissue samples in the TCGA-THCA dataset (Fig. 3b). Therefore, *PIK3AP1* was selected as the gene directly targeted by miR-1246 for further dual-luciferase analysis. Thus, WT and mutant PIK3AP1 reporter gene vectors (WT-PIK3AP1 and mut-PIK3AP1) were prepared, with the later containing 7 bp mutations at the predicted miR-1246 binding site (Fig. 3c). These miR-1246 mimics and vectors were co-transfected into HEK293 cells prior to an analysis of luciferase activity. As shown in Fig. 3d, miR-1246 mimics remarkably suppressed the luciferase activity of cells transfected with WT luciferase reporter gene vectors, while miR-1246 inhibitors amplified it. Mutations at the putative miR-1246 binding sites eliminated changes in luciferase activity attributable to miR-1246 overexpression, confirming that miR-1246 directly targets PIK3AP1. Next, western blotting was used to detect PIK3AP1, p-PI3K, PI3K, p-AKT and AKT levels in TPC-1 and FTC-133 cells. The results showed that miR-1246 mimics decreased the protein level of PIK3AP1 and PI3K/AKT phosphorylation (Fig. 3e).

### miR-1246 function is negatively correlated to that of PIK3AP1

**Fig. 4:**
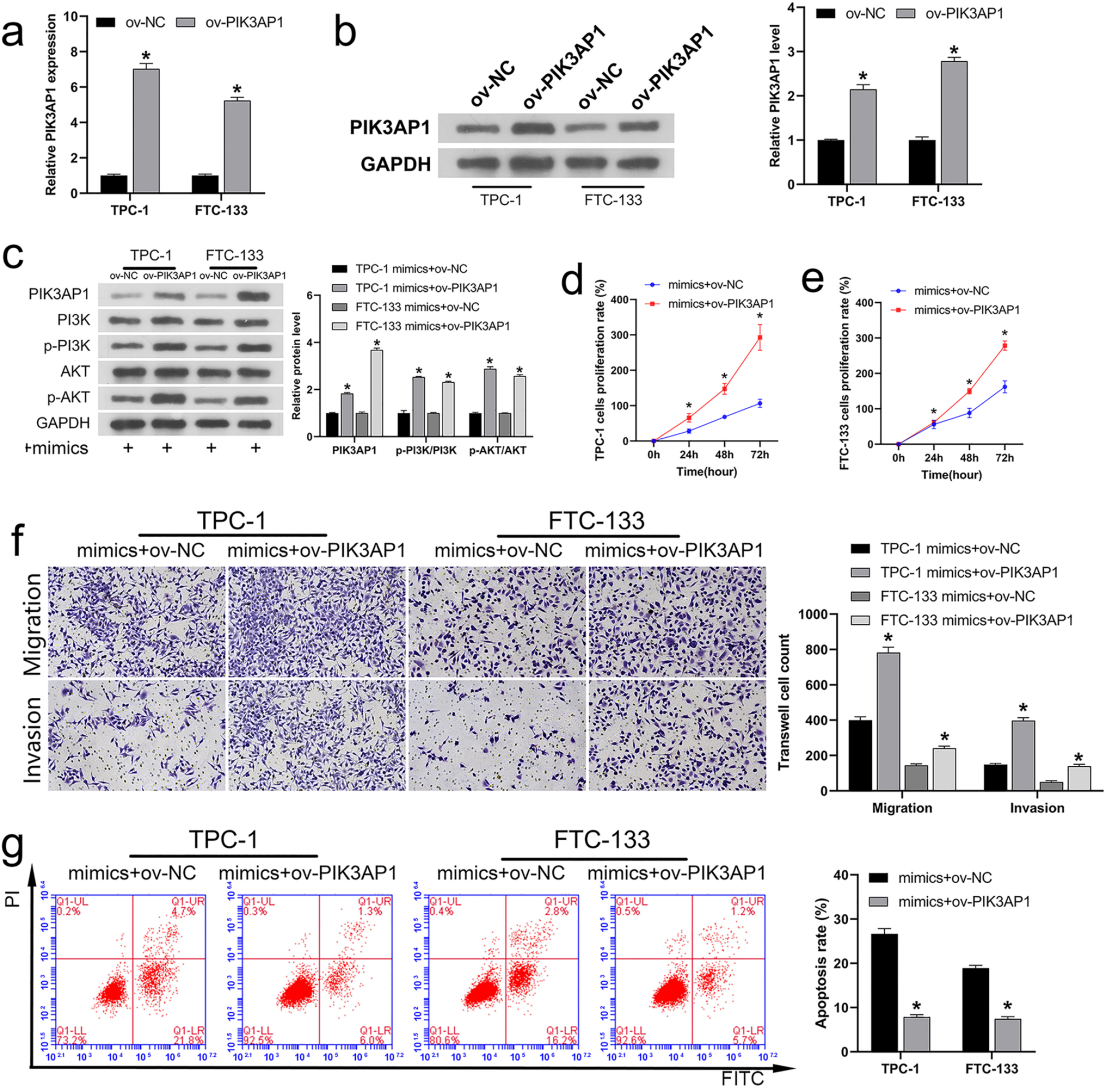
MiR-1246 is a negative regulator of PIK3AP1. **a** The expression of PIK3AP1 in ov-PIK3AP1-trasfected TPC-1 and FTC-133 cells detected utilizing RT-qPCR. **b** Western blotting assessment of PIK3AP1 protein levels in ov-PIK3AP1-trasfected TPC-1 and FTC-133 cells. Following simultaneous co-transfection with miR-1246 mimics and ov-PIK3AP1: **c** Western blotting analysis of the protein levels of PIK3AP1, p-PI3K, PI3K, p-AKT, and AKT in TPC-1 and FTC-133 cells. **d**, **e** CCK-8 analysis of the rate of proliferation for TPC-1 and FTC-133 cells. **f** Transwell analysis of the invasion and migration of TPC-1 and FTC-133 cells (magnification, 200×). **g** Apoptosis levels of TPC-1 and FTC-133 cells analyzed utilizing FCM. *P < 0.05. *PIK3AP1* phosphoinositide 3-kinase adapter protein 1, *ov-PIK3AP1* PIK3AP1 overexpression plasmid, *RT-qPCR* Real-time quantitative reverse transcription polymerase chain reaction, *FCM* flow cytoMetry, *ov* overexpression, *CCK-8* cell counting kit-8, *NC* negative control

To further confirm the negatively correlated between miR-1246 and PIK3AP1, we first constructed and verified a PIK3AP1 overexpression vector. RT-qPCR and western blotting exhibited that the gene expression (Fig. 4a) and protein level (Fig. 4b) of PIK3AP1 were significantly upregulated in both cell lines after transfection of the PIK3AP1 overexpression plasmid (ov-PIK3AP1 plasmid), indicating that the overexpression vector was successfully constructed. Then, ov-NC or ov-PIK3AP1 plasmids were co-transfected into the cells of TPC-1 and FTC-133 with miR-1246 mimics. Western blotting results showed that PIK3AP1 overexpression reversed the protein transcription of PIK3AP1 induced by miR-1246 mimics and the phosphorylation of PI3K/AKT in TPC-1 and FTC-133 cells (Fig. 4c). Analysis of CCK-8 (Fig. 4d, e), migration and invasion (Fig. 4f), and FCM (Fig. 4g) results showed that PIK3AP1 overexpression also reversed the decreased the rate of cell proliferation, invasion and migration ability and increased apoptosis induced by miR-1246 mimics. In conclusion, miR-1246 is a negative regulator of PIK3AP1.

### miR-1246 modulates PI3K/AKT signaling by targeting PIK3AP1

**Fig. 5:**
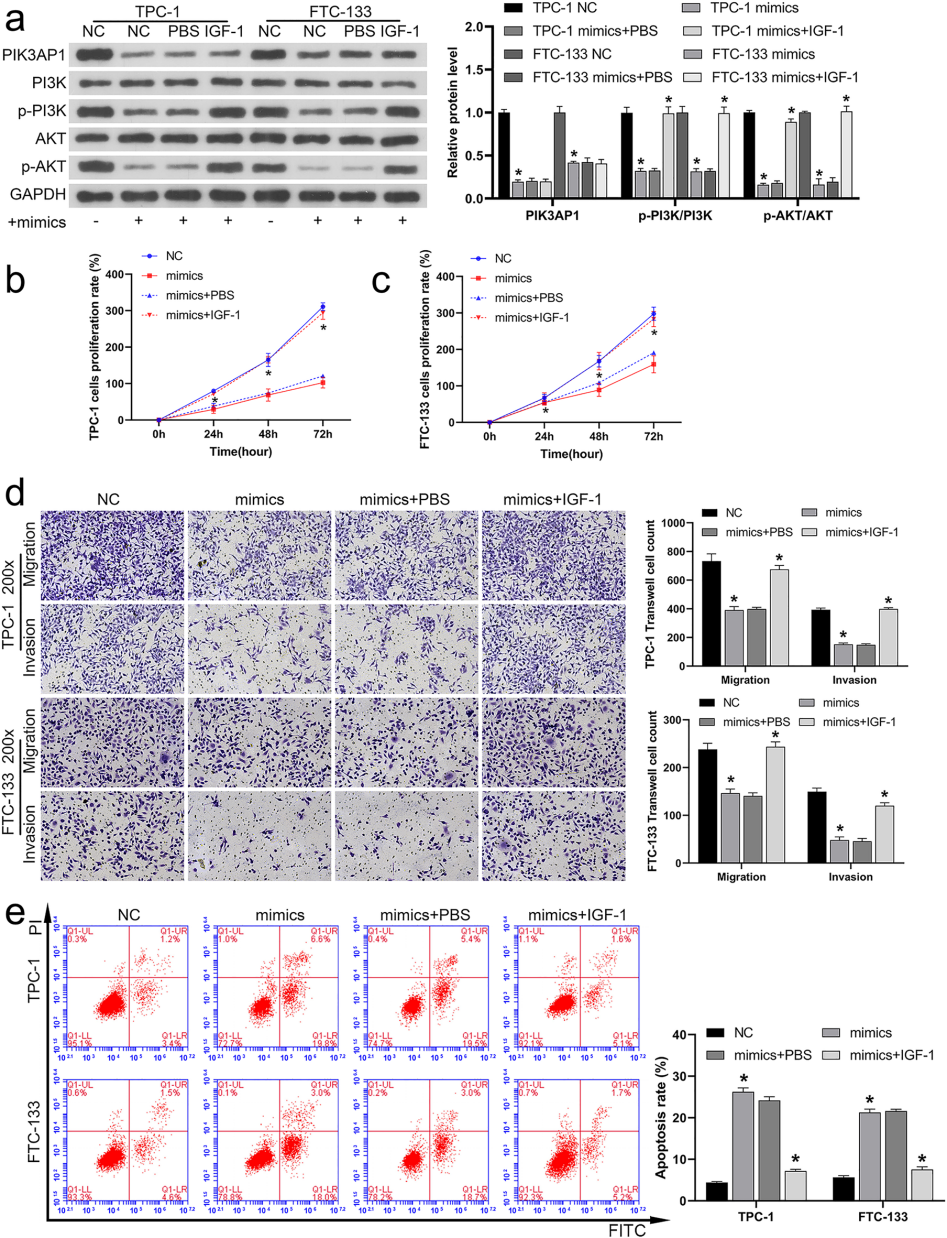
miR-1246 modulates PI3K/AKT signaling by targeting PIK3AP1. IGF-1, an activator of the signaling pathway of PI3K/AKT, was added at the same time of miR-1246 transfection. **a** Levels of protein for PIK3AP1, p-PI3K, PI3K, p-AKT, and AKT in TPC-1 and FTC-133 cells detected using Western blotting. **b**, **c** CCK-8 analysis of TPC-1 and FTC-133 proliferation. **d** Transwell analysis of the migration and invasion ability of these cells (magnification, 200×). **e** Apoptosis levels of these cells analyzed using FCM. *P < 0.05. *PIK3AP1* phosphoinositide 3-kinase adapter protein 1, *PI3K/AKT* phosphoinositol 3-kinase/protein kinase B, *IGF-1* insulin growth factor-1, *CCK-8* cell counting kit-8, *FCM* Flow cytometry, *NC* negative control

Previously, we demonstrated that miR-1246 mimics inhibit PI3K/AKT phosphorylation. Here, we investigated whether the effects of miR-1246 mimics could be reversed by activating PI3K/AKT signaling. Therefore, while transfecting cells with miR-1246 mimics, we added IGF-1, an activator of the signaling pathway of PI3K/AKT. Next, western blotting was used to detect PIK3AP1, PI3K, p-PI3K, AKT and p-AKT levels in the cells of TPC-1 and FTC-133. As demonstrated in Fig. 5a, the inhibiting effect of miR-1246 mimics on PI3K/AKT phosphorylation was reversed by IGF-1. Notably, miR-1246 mimics inhibited the protein level of PIK3AP1, which was not significantly affected by IGF-1 addition (*P* > 0.05). Meanwhile, CCK-8 (Fig. 5b, c), migration and invasion (Fig. 5d) and FCM (Fig. 5e) results revealed that the reduced proliferation rate, migration and invasion ability of miR-1246 mimics and the promotion of cell apoptosis were also reversed by IGF-1. These results suggested that miR-1246 suppressed the activity of the PI3K/AKT pathway via targeting PIK3AP1.

### miR-1246 inhibited the growth of tumor and lung metastasis in vivo

**Fig. 6:**
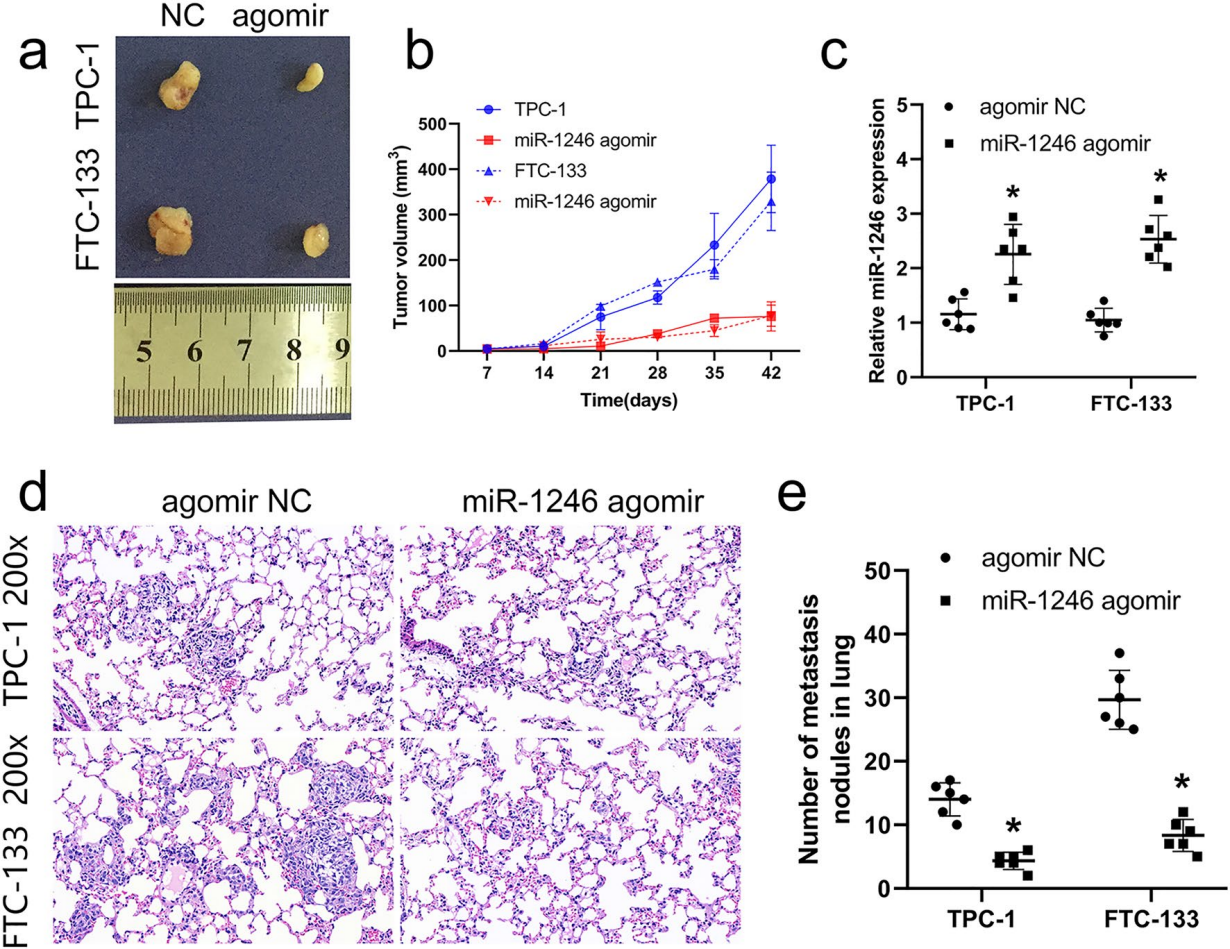
Overexpression of miR-1246 inhibits the growth of tumors and metastasis in lung tissues. After transfection of TPC-1 and FTC-133 cells with miR-1246 agomir, bred for 6 weeks and then sacrificed, subcutaneous tumorigenesis and lung metastasis assays (caudal vein injection) were performed. **a** Image of the tumor 6 weeks after subcutaneous xenograft. **b** Calculation of tumor volume. **c** The expression of miR-1246 in tissues of the tumor was analyzed through RT-qPCR. **d** HE staining results of lung tissue (200×). **e** Calculation of tumor nodules in lung tissue. The groups are as follows: TPC-1-miR-1246 agomir, TPC-1-miR-1246 agomir NC, FTC-133-miR-1246 agomir, and FTC-133-miR-1246 agomir NC groups. *P < 0.05. *NC* negative control

To further affirm the effect of miR-1246 on the cells of thyroid cancer, we transfected miR-1246 agomir and negative control agomir NC in the cells of TPC-1 and FTC-133. Transfected cells were injected into mice to establish subcutaneous tumorigenesis and caudal vein models. Tumor imaging and RT-qPCR results showed that miR-1246 agomir inhibited tumor growth (Fig. 6a, b) and expression of miR-1246 was upregulation in agomir group compared with NC group (Fig. 6c). The maximum tumor diameter observed for a single subcutaneous tumor during the xenograft assay was 1.14 cm. Lung HE results showed that relative to the NC group, miR-1246 agomir significantly inhibited the lung metastasis (Fig. 6d) and tumor nodules (Fig. 6e) in vivo. The above results confirm that miR-1246 inhibits in vivo tumor growth and metastatic progression.

## Discussion

In the past few decades, miRNA dysregulation has been reported to be related to oncogenesis and cancer progression [28, 29]. Studies have confirmed that the GEO database can mine a large number of miRNAs upregulated and downregulated in thyroid cancer [30], these malregulated miRNAs, such as miR-221-3p and miR-7-5p [31, 32], are closely associated with the progression of thyroid cancer. Among them, High or low miR-1246 expression in cancer and its role in cancer development have been widely reported [33–35]. We searched for abnormally regulated miRNAs in the GEO database and found that miR-1246 was significantly reduced in thyroid cancer relative to healthy tissue. Notably, how miR-1246 affects thyroid cancer has not been reported previously, it makes miR-1246 a potential new target for thyroid cancer treatment. Importantly, miR-1246 was one of the 26 miRNAs that met the *P* < 0.01 and log_2_|Fc|> 1.5 criteria, suggesting a potential role of under-expressed miR-1246 in thyroid cancer pathogenicity.

It has been reported that miR-1246 is related to the proliferation, apoptosis, migration, and invasion of cancer cells. In lung cancer, miR-1246 promoted the epithelial–mesenchymal transition of A549 cells and improved the migration and invasion ability of lung cancer epithelial cells [36]. Du et al. [37] demonstrated that inhibition of miR-1246 inhibited the growth of cervical cancer tumors and promoted apoptosis of SiHa cells. Wang et al. [38] found that overexpressing miR-1246 can induce proliferation, invasion, and migration of colorectal cancer cells. Herein, we found that miR-1246 overexpression significantly inhibited thyroid cancer cell proliferative, migratory, and invasive activity while promoting apoptotic death, in line with prior reports in other cancer types.

miRNA–mRNA interactions can directly influence tumorigenesis [39–41]. To better explore the mechanisms involved in thyroid cancer, the putative target genes of miR-1246 were explored in the TargetScan database, and the upregulated mRNAs were analyzed in combination with the TCGA-THCA database data. PIK3AP1 was found to be a possible = transcription target of miR-1246. More importantly, KEGG analysis of PIK3AP1 showed that it is one of the genes affecting PI3K/AKT signaling. However, whether miR-1246 can regulate PI3K/AKT signaling by targeting PIK3AP1 requires further investigation. Our study showed that overexpression of miR-1246 could inhibit the level of protein related to PIK3AP1 and the phosphorylation of PI3K/AKT, inhibit the proliferation of cells, invasion and migration, and improve cell apoptosis. This result was reversed by overexpression of PIK3AP1, confirming the negative regulatory relation between miR-1246 and PIK3AP1. Subsequently, we further explored the association between miR-1246 and PI3K/AKT signaling. Our data suggest that activation of PI3K/AKT signaling reverses the miR-1246-induced inhibition of this signaling pathway and the developmental arrest of cell function. The results confirmed that miR-1246 regulates PI3K/AKT signal transduction by targeting PIK3AP1, thus affecting the proliferation of thyroid cancer cells.

To further confirm these findings, subcutaneous tumorigenesis and lung metastases were evaluated in vivo. The achievements illustrated that the overexpressing miR-1246 can hinder the growth of tumor and metastasis in lung tissues.

## Conclusion

miR-1246 hinders the proliferation and the growth of tumors related to the thyroid cancer cells in vivo through regulating PIK3AP1 and inhibiting the signaling of PI3K/AKT. miR-1246 is a modern biomarker and treatment target for thyroid cancer. These data offer a valuable foundation for future studies of the pathological basis for thyroid cancer.

## Data Availability

The microarray data (GSE53072_RAW.tar) referenced in the study are available in a public repository on the GEO website (http://www.ncbi.nlm.nih.gov/geo). All other data supporting the findings of this study are available in the manuscript, as well as from the corresponding authors upon reasonable request.

## Code availability

All data codes supporting the results of this study can be provided by the corresponding author, if required.
